# TGF-β2 induces Grb2 to recruit PI3-K to TGF-RII that activates JNK/AP-1-signaling and augments invasiveness of *Theileria*-transformed macrophages

**DOI:** 10.1038/srep15688

**Published:** 2015-10-29

**Authors:** Malak Haidar, Jessie Whitworth, Gaelle Noé, Wang Qing Liu, Michel Vidal, Gordon Langsley

**Affiliations:** 1 Laboratoire de Biologie Cellulaire Comparative des Apicomplexes, Faculté de Médicine, Université Paris Descartes - Sorbonne Paris Cité, France; 2 Inserm U1016, Cnrs UMR8104, Cochin Institute, Paris, 75014 France; 3 UF Pharmacocinétique et pharmacochimie Hôpital Cochin, Paris, France Assistance Publique Hôpitaux de Paris; 4 UMR8638 CNRS, Faculté de Pharmacie, Université Paris Descartes, PRES Sorbonne Paris Cité, Paris, France

## Abstract

*Theileria*-infected macrophages display many features of cancer cells such as heightened invasive capacity; however, the tumor-like phenotype is reversible by killing the parasite. Moreover, virulent macrophages can be attenuated by multiple *in vitro* passages and so provide a powerful model to elucidate mechanisms related to transformed macrophage virulence. Here, we demonstrate that in two independent *Theileria*-transformed macrophage cell lines Grb2 expression is down-regulated concomitant with loss of tumor virulence. Using peptidimer-c to ablate SH2 and SH3 interactions of Grb2 we identify TGF-receptor II and the p85 subunit of PI3-K, as Grb2 partners in virulent macrophages. Ablation of Grb2 interactions reduces PI3-K recruitment to TGF-RII and decreases PIP3 production, and dampens JNK phosphorylation and AP-1-driven transcriptional activity down to levels characteristic of attenuated macrophages. Loss of TGF-R>PI3-K>JNK>AP-1 signaling negatively impacts on virulence traits such as reduced JAM-L/ITG4A and Fos-B/MMP9 expression that contribute to virulent macrophage adhesion and invasiveness.

*Theileria annulata* is an intracellular protozoan and member of the phylum *Apicomplexa*. *Theileria* parasites (*T. annulata* and *T. parva*) are unusual eukaryotes in that they possess the property of being able to transform their host cells. Transformed leukocytes display many characteristics of tumors such as uncontrolled proliferation independent of exogenous growth factors, resistance to apoptosis, and an increased ability to disseminate and invade organs *in vivo*^1^,^2^. Moreover, virulence of transformed macrophages is reversed upon drug-induced parasite death^3^,^4^ and attenuated by multiple *in vitro* passages^5^.

In regions endemic for tropical theileriosis indigenous breeds of cattle (ex: *Bos indicus* also known as Sahiwals) are more resistant to disease, exhibit less symptoms and recover from an infectious dose, which is often fatal to non-indigenous breeds (ex: *B. taurus* also known as Holstein-Friesian—HF)^6^. Nonetheless, no great difference in infection-induced expression levels of pro-inflammatory factors such as IL-6^7^, GM-CSF^8^ and TNF^9^ was detected between disease-resistant and susceptible cattle. By contrast, induction of TGF-β2 by *T. annulata* infection is greater in macrophages isolated from disease-susceptible (HF) compared to disease-resistant (Sahiwals) cattle and this correlates with the higher invasive capacity of HF-infected macrophages^10^. Upon attenuation *in vitro*, the HF cell line displayed both reduced TGF-β2 expression and invasiveness, which was re-established by exogenous TGF-β2^10^. The ensembles of these observations are consistent with a pro-metastasis role of TGF-β2 in virulent *Theileria*-infected macrophages.

Transforming growth factor-β (TGF-β) is a family of cytokines known to regulate cell growth, differentiation and motility^11^. TGF-β1 and TGF-β3 can bind with high affinity to the TGF-β type II receptor (TGF-RII), which leads to the recruitment of the type I receptor (TGF-RI). The constitutively active TGF-RII serine/threonine kinase phosphorylates and activates the TGF-RI receptor kinase, which leads to the recruitment and activation of Smad2 and Smad3. Activated Smad2 and Smad3 then bind to Smad4, and the entire complex translocates to the nucleus to activate downstream target genes^12^. While type II receptor binds with low affinity to TGF-β2, type III receptor (TGF-RIII) binds with high affinity to all three isoforms and is needed to present TGF-β2 to TGF-RII^13^. Besides the canonical Smad pathway, many other pathways such as JNK^14^, Ras^14^, PI3-K^15^ and PKA^16^ are also regulated by TGF-β. This could explain why TGF-β signaling pathways play such a complex role in mediating cellular processes.

Phosphatidylinositol-4, 5-bisphosphate 3-kinases (PI3-K) is a family of signal transducing enzymes that are also involved in cell growth, proliferation, motility and survival^17^. The PI3-K family is divided into 3 classes based on their primary protein structure, regulation and *in vitro* lipid substrate specificity^18^. Class I PI3-kinases are made up of a p110 catalytic subunit and a p85 regulator subunit, which harbors an SH3 domain. PI3-kinases are capable of phosphorylating the 3-position hydroxyl group of the inositol ring of phosphatidylinositol to form phosphatidylinositol biphosphate (PIP_2_) and phosphatidylinositol triphosphate (PIP_3_) and other 3-phosphorylated phosphoinositides. This group of signaling kinases contains phosphoinositide-binding domains that are recruited for binding to cell membranes. Both we, and others have previously demonstrated that *T. parva* and *T. annulata*-infected B-lymphocytes rely on constitutively activated PI3-K for their continued proliferation^8^,^19^,^20^.

PI3-K also acts as an upstream regulator of the c-Jun NH_2_-terminal kinase (JNK)^8^,^21^. JNK is constitutively activated in *Theileria*-infected cells^22^,^23^. Proteins of the Jun and Fos families combine to form the heterodimeric AP-1 transcription factor, which enters the nucleus and binds to specific DNA sequences to modulate target gene expression. AP-1 activity is induced by stimuli such as growth factors and cytokines that bind to specific cell surface receptors^24^. AP-1 is a key transcription factor induced in virulent *Theileria*-transformed leukocytes^23^ and AP-1-driven transcription underpins the hyper-dissemination virulence phenotype^25^.

The Growth factor receptor-bound protein 2 (Grb2) is an adaptor used to recruit accessory proteins in signal transduction pathways^26^. Grb2 contains three domains; one Src Homology 2 (SH2) and two Src Homology 3 (SH3) domains at the N- and C-termini^27^,^28^. The SH2 domain participates in signal transduction in tyrosine kinase pathways by recognizing specific amino acids motifs in partner proteins, whereas SH3 domains recognize proline-rich sequences within specific partners. The two SH3 domains form a direct complex with the proline-rich regions of the partner proteins, while the SH2 domain binds to tyrosine-phosphorylated motifs in receptors^29^.

We have previously demonstrated that heightened PI3-K activity is essential for the proliferation of *Theileria*-transformed B cells^8^ and have shown that one mechanism by which PI3-K mediates proliferation of infected B lymphocytes is through induction of a granulocyte-monocyte colony-stimulating factor (GM-CSF)-dependent autocrine loop^8^. Moreover, we have shown that the level of TGF-β2 is augmented in virulent macrophages and this led us to test the role of TGF-β2 in activation of the PI3-K signaling pathway^10^. We now describe how loss of Grb2 expression ablates PI3-K recruitment to TGF-RII and how a TGF-β2/Grb2/PI3-K/AP-1 pathway contributes to the hyper-invasive virulence phenotype of *Theileria*-transformed macrophages.

## Results

### Grb2 is a TGF-β2-target in *Theileria*-transformed macrophages

**Figure 1:**
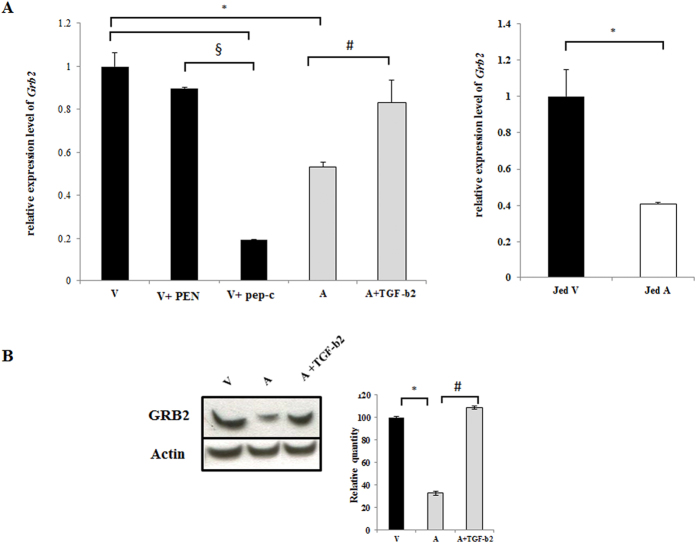
Grb2 is a TGF-β2-target gene in *Theileria*-infected macrophages. (**A**) Total RNA was extracted from virulent (V) and attenuated (A) macrophages. Relative mRNA levels of the different genes were determined by qPCR and normalized to *GAPDH* transcripts. **Left panel.** The transcription level of *Grb2* is higher in virulent macrophages compared to attenuated ones. The treatment with peptidimer-c decreases the expression level of *Grb2* in virulent macrophages, whereas adding exogenous TGF-β2 to attenuated macrophages restores *Grb2* expression. **Right panel.** Levels of *Grb2* expression in Jed4 macrophages. (**B**) Western blot analysis with anti-Grb2 antibody using a whole cell lysates (10 μg of protein) derived from virulent and attenuated macrophages treated with TGF-β2 or not. *Grb2* is diminished in attenuated macrophages and stimulation by TGF-β2 of attenuated macrophages restores protein levels compared to virulent Ode. *Grb2* protein levels were normalized to *β*-actin protein levels. *p < 0.05 compared to virulent macrophages, #p < 0.05 compared to attenuated macrophages, ^§^p < 0.05 compared to virulent macrophages treated with PEN.

Grb2 is implicated in many cellular functions including adhesion. We have previously shown that *Theileria*-infected Ode macrophages that display a heightened capacity of adhesion typical of virulent disease-causing infected macrophages compared to live vaccine lines used to treat tropical theileriosis that are attenuated for this virulence trait^30^. So, we checked the expression of *Grb2* in virulent and attenuated macrophages by qPCR analysis (Fig. 1A). In Fig. 1A one can observe an attenuation-associated 50% decrease in *Grb2* expression and the decrease was restored by stimulating attenuated macrophages with exogenous TGF-β2. When virulent macrophages are treated with peptidimer-c transcription of *Grb2* is reduced by approximately 80%. Furthermore, as TGF-β2 is also down regulated in attenuated Jed4 macrophages isolated from an infected animal in Tunisia^30^ it follows that *Grb2* expression is also diminished (Fig. 1A, right panel). Changes in transcription of *Grb2* were reflected at the protein level (Fig. 1B), as an approximate 40% decline in Grb2 protein was observed in attenuated macrophages and protein levels are restored by exogenous TGF-β2. Taken together; the results show that TGF-β2-signaling is responsible for high levels of Grb2 in virulent *Theileria*-infected macrophages of different geographical origins and ablation of Grb2 accompanies loss of *Theileria*-transformed macrophage virulence.

### Grb2 associates with TGF-RII and the p85 subunit of PI3-K

**Figure 2:**
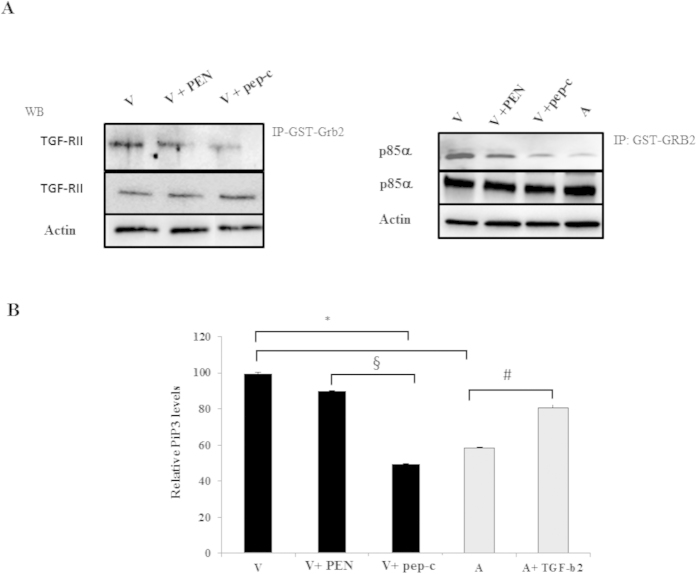
Grb2 associates with TGF-RII and the p85 subunit of PI3-K. (**A**) **Left panel.** Pull-down assay performed with TGF-RII receptor expressed by *Theileria*-transformed macrophages incubated with GST-Grb2 beads. Bottom two rows demonstrate equivalent amounts of actin and TGF-RII in the cell extracts mixed with the GST-Grb2 beads. The top row demonstrates more TGF-RII is bound to GST-Grb2 in virulent macrophages and binding is not diminished by PEN treatment. TGF-RII binding is ablated when Grb2 SH3-interactions are dampened by treatment with peptidimer-c. **Right panel.** Immunoprecipitation analysis with anti-p85 antibody using whole cell lysates derived from virulent macrophages (V) incubated with GST-Grb2 beads overnight. Bottom two rows indicate that equivalent amounts of cell extract containing p85α and actin were mixed with GST-Grb2 beads. Top row shows the amount of p85α bound to GST-Grb2 beads in virulent and attenuated macrophages. Treatment of virulent macrophages with PEN did not diminish the amount of p85α bound to GST-Grb2. Ablation of SH3 domain interactions of Grb2 with peptidimer-c in virulent macrophages diminishes Grb2 binding of p85α to attenuated levels. (**B**) Ablating Grb2 partner-interactions with peptidimer-c reduces PI3-K activity to attenuated levels. PI3-K activity in virulent macrophages is decreased when virulent macrophages are treated with 1 μM of peptidimer-c, and adding exogenous TGF-β2 to attenuated macrophages increases PI3-K activity. *p < 0.05 compared to virulent macrophages, #p < 0.05 compared to attenuated macrophages, §p < 0.05 compared to virulent macrophages treated with PEN.

We examined whether Grb2 is an upstream player in the PI3-K signaling pathway essential for *Theileria*-infected leukocyte proliferation by using peptidimer-c to see if the PI3-K levels were decreased after ablation of SH3 interactions, as peptidimer-c specifically recognizes SH3 domains of Grb2^29^,^31^. Firstly, we examined if there was an association between Grb2 and the p85 subunit, and if there was a difference in their interaction between the virulent and attenuated cell lines would treatment of virulent macrophages with peptidimer-c ablate the interaction to levels observed for attenuated macrophages. To detect interactions between Grb2 and p85 pull-downs using GST-Grb2 beads were performed (Fig. 2A). The pull-down demonstrated an interaction between Grb2 and p85 in *Theileria*-infected macrophages and revealed a dampened association in attenuated macrophages. The association between Grb2 and p85 was ablated by peptidimer-c that competes for Grb2 SH3 domain binding to partners (Fig. 2A, left panel). Thus, the interaction with p85 occurs via the SH3 domains of Grb2 and this can be blocked by peptidimer-c.

Grb2 is known to associate with TGF-RII following receptor phosphorylation at Tyr284 by Src^32^. GST-Grb2 pull-downs were therefore also used to demonstrate an interaction between Grb2 and TGF-RII in *Theileria*-infected macrophages. Again, the association was diminished upon treatment of virulent macrophages with peptidimer-c (Fig. 2A, right panel). Taken all together, one suggests that Grb2 recruits the regulatory p85 subunit of PI3-K to TGF-RII in *Theileria*-infected macrophages.

### p85 recruitment to TGF-RII by Grb2 stimulates PI3-K activity

Having demonstrated that the Grb2 and p85 interaction is ablated by peptidimer-c it follows that peptidimer-c treatment should dampen PI3-K activity, as p85 recruits the catalytic p110 subunit proximal to the plasma membrane, where it converts PIP_2_ to PIP_3._ The level of PIP_3_ therefore is a reflection of PI3-K activity. Treating virulent macrophages with peptidimer-c reduced PI3-K activity to that of attenuated macrophages (Fig. 2B). Importantly, TGF-β2 stimulation of attenuated macrophages restores host cell PIP_3_ to virulence levels demonstrating that Grb2 recruitment of p85 to TGF-RII augments PI3-K activity.

### Recruitment of p85 to TGF-RII by Grb2 promotes JNK phosphorylation and AP-1 activation

**Figure 3:**
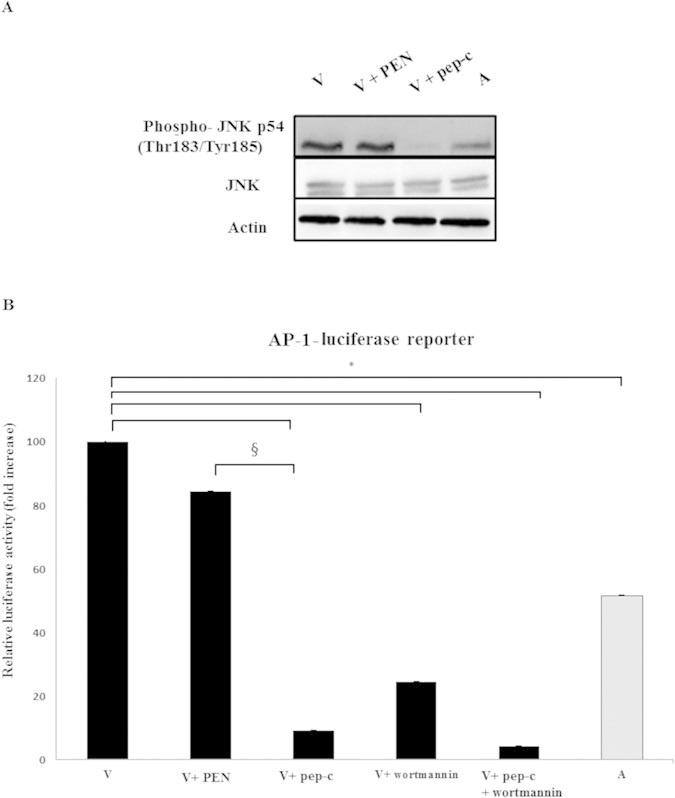
JNK phosphorylation and AP-1 activity are affected by ablation of Grb2 interactions. (**A**) **Top panel.** JNK phosphorylation is dampened in virulent macrophages treated with peptidimer-c compared to non-treated virulent macrophages. Treatment with the penetrating peptide PEN had no effect. (**B**) **Bottom panel.** AP-1 luciferase activity in virulent macrophages is decreased upon treatment with 1μM of peptidimer-c. Virulent macrophages were also treated with the PI3-K inhibitor Wortmannin. When attenuated macrophages were stimulated with 5 ng/ml of TGF-β2, AP-1 luciferase activity returned to virulent levels. Luciferase and β-galactosidase activities were measured as described in Methods and for each independent transfection relative luciferase activity was normalized to the corresponding β-galactosidase activity. *p < 0.05 compared to virulent macrophages, ^§^p < 0.05 compared to virulent macrophages treated with PEN.

Activator Protein 1 (AP-1) is constitutively induced via PI3-K-signaling in *Theileria*-infected leukocytes^8^. AP-1 activity is required for the survival of *T. parva*-transformed B cells^23^ and for dissemination of *T. annulata*-transformed macrophages^25^. It follows ablation of the Grb2-p85-TGF-RII association and consequent drop in PI3-K activity should be reflected in decreased JNK phosphorylation and AP-1-mediated transcription, and this was confirmed by western blot analysis of JNK phosphorylation and by measuring AP-1-driven luciferase activity (Fig. 3). Treatment of virulent macrophages with peptidimer-c ablates JNK phosphorylation, and when virulent macrophages are treated with peptidimer-c the loss of PI3-K activity is comparable to the loss observed with the specific PI3-K inhibitor Wortmannin (Fig. 3).

### Ablation of SH3 interactions of Grb2 dampens AP-1 transactivation and impacts on cellular adhesion and invasion

**Figure 4:**
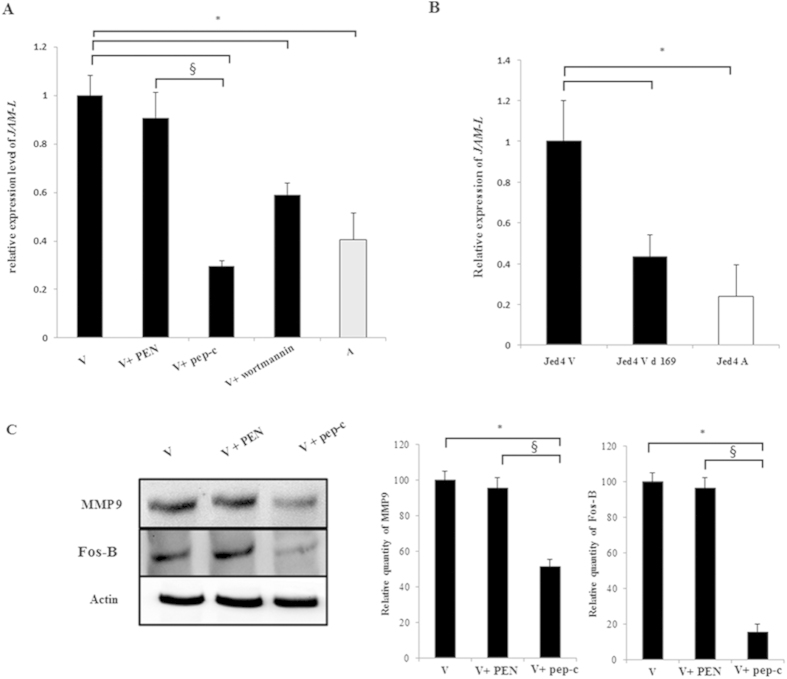
JAM-L is a downstream adhesion effector of Grb2/PI3K/AP-1 signaling. Total RNA was extracted from virulent, engineered (delta169) virulent and attenuated Jed4 macrophages. Relative mRNA levels for *jam-l* were determined by qPCR and normalised to *GAPDH* transcripts. (**A**) *Jam-l* is transcriptionally up regulated in virulent compared to attenuated macrophages. Inhibition of Grb2 interactions and PI3-K signaling in virulent macrophages decreases the level of *jam-l*. (**B**) The level of *jam-l* transcription in engineered (delta169) Jed4 macrophages is reduced to that of attenuated Jed4 macrophages. (**C**) The protein expression levels of Fos-B and MMP9 in virulent macrophages is downregulated upon treatment with peptidimer-c. Fos-B and MMP9 protein levels were normalized to β-actin protein level. *p < 0.05 compared to virulent macrophages, ^§^p < 0.05 compared to virulent macrophages treated with PEN.

Our focus turned to JAM-L, as it can bind to CAR and recruit PI3-K^33^. Since we have demonstrated above that Grb2 activates PI3-K/AP-1 signaling and knowing that Grb2 can regulate cellular adhesion^34^, we asked if Grb2 regulates adhesion of *Theileria*-infected macrophages through JAM-L, as anti-JAM-L antibodies dampen adhesion of *Theileria*-infected macrophages^30^. So we used peptidimer-c to ablate the SH3 domain interactions of Grb2 and looked at JAM-L expression and adhesion capacity in *Theileria*-infected macrophages. By qPCR there was an almost 60% decrease in *jam-l* expression indicating that upon attenuation transcriptional *jam-l* declines (Fig. 4A). Treatment of virulent macrophages with peptidimer-c, or a specific inhibitor of PI3-K (Wortmannin) also decreased *jam-l* transcription.

To link *jam-l* expression to AP-1-mediated transcription, we exploited a *Theileria*-transformed macrophage cell line of Tunisian origin, where AP-1-driven transcription had been handicapped by engineered expression of a dominant-negative mutant (Δ169) of c-Jun^25^. In the engineered cell line (virulent delta169) *jam-l* mRNA levels drop to those typical of attenuated macrophages demonstrating that expression of *jam-l* is AP-1-dependent (Fig. 4B). Since *jam-l* transcription decreases upon attenuation and peptidimer-c treatment ablates *jam-l* expression in virulent macrophages, we examined the ability of peptidimer-c to dampen transformed macrophage adhesion.

The Matrix MetalloProteinase (MMP) 9 plays a central role in tumor progression and metastasis by stimulating cell migration, tumor invasion, and angiogenesis^35^. Consistently, MMP9 functions as a mediator of metastasis of *Theileria*-transformed leukocytes^36^,^37^. Moreover, *mmp9* expression is driven by the transcription factor AP-1 and differences in *mmp9* transcription between virulent and attenuated *Theileria*-transformed macrophages is due to differential recognition of the AP-1 motif in the *mmp9* promoter^38^. Significantly, a fast migrating AP-1 family member failed to bind to the *mmp9* promoter in attenuated macrophages^38^. By its size the fast migrating species could be Fos-B, a member of a family of transcription factors made up of Fos, Fos-B, Fos-L1 and Fos-L2 that are leucine zipper proteins, which dimerize with members of the Jun family to form different AP-1 heterodimers^39^. As ablation of SH3 domain interactions of Grb2 diminishes transactivation of AP-1 (Fig. 3), we examined the expression levels of both Fos-B and MMP9 and both were diminished following peptidimer-c treatment of virulent macrophages (Fig. 4).

### Ablation of p85 recruitment by Grb2 decreases adhesion and invasiveness of *Theileria*-infected macrophages

**Figure 5:**
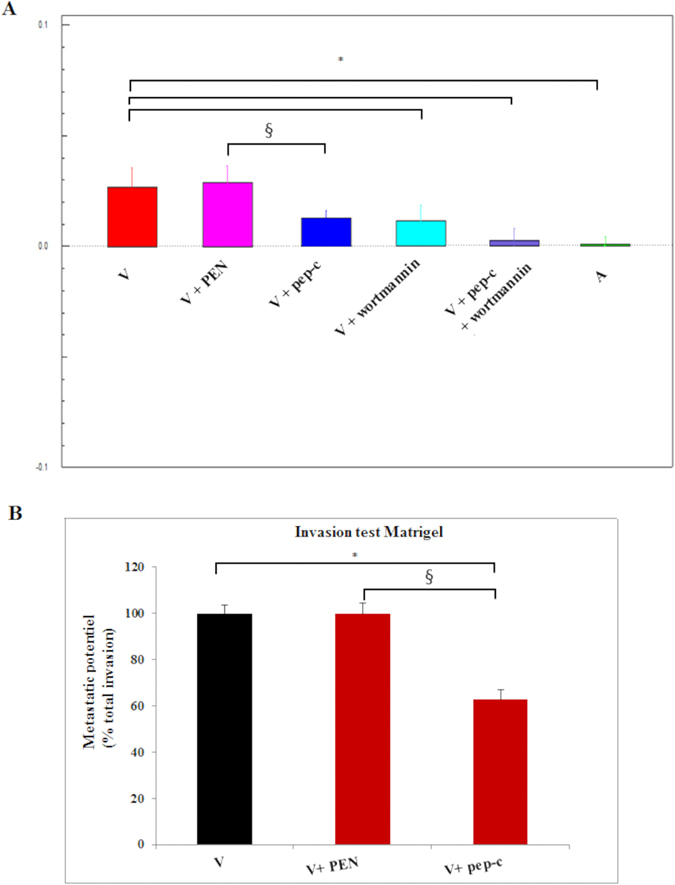
Ablation of SH3 domain interactions of Grb2 diminishes adhesion to fibronectin and Matrigel traversal. (**A**) Adhesion to fibronectin decreases to attenuated levels when virulent macrophages are treated with peptidimer-c, or Wortmannin. Virulent macrophages were treated with 1 μM of peptidimer-c and/or 100 nM of Wortmannin. Adhesion to fibronectin was then measured. (**B**) Matrigel traversal of virulent *Theileria*-transformed macrophages. Blockade of SH3 domain interactions of Grb2 by peptidimer-c treatment decreased Matrigel traversal of virulent macrophages. No effect was observed upon treatment with PEN. *p < 0.05 compared to virulent macrophages, ^§^p < 0.05 compared to virulent macrophages treated with PEN.

In order to confirm the role of Grb2 in promoting adhesion of virulent *Theileria*-infected macrophages, we performed a dynamic *in vitro* adhesion assay. Peptidimer-c treatment reduces adhesion of virulent macrophages to attenuated levels (Fig. 5A). Wortmannin treatment had a similar affect consistent with adhesion being regulated by a Grb2>PI3-K signaling pathway. In parallel, we performed *in vitro* invasion (Matrigel chamber) assays. The capacity of virulent macrophages to traverse Matrigel is significantly decreased when virulent macrophages are treated with peptidimer-c (Fig. 5B). No effect was observed when treated with the penetrating peptide PEN used as a negative control.

## Discussion

We targeted Grb2 because it is an adaptor protein that facilitates formation of large signaling complexes that play pivotal roles in receptor-mediated signal transduction, an example is the association of Grb2 with TGF-RII following receptor phosphorylation on Tyr284 by Src^32^. Additionally, there’s a significant difference in TGF-β2 expression between the virulent and attenuated *Theileria*-infected macrophages^10^. For these reasons we dissected a possible Grb2/TGF-RII association by ablating Grb2 SH3 interactions by peptidimer-c treatment^26^. We found that not only does Grb2 interact with TGF-RII, but that *Grb2* is also a TGF-β2-target gene in *Theileria*-transformed macrophages. As we have shown that ablating Grb2 SH3 interactions dampens AP-1-driven luciferase it suggests that *Grb2* is also an AP-1-target gene and possibly even a CREB-target gene^30^. Multiple binding partners have been described for Grb2 including the regulatory p85 subunit of PI3-K^40^. Firstly, we found that TGF-β2 stimulation increased PI3-K activity, whilst ablation of SH3 interactions of Grb2 decreased its activity. Then, we confirmed using GST-Grb2 pull downs that TGF-β2 stimulation of PI3-K activity was facilitated by Grb2; the decrease in association between Grb2 and the p85 subunit in peptidimer-c treated cells confirmed that Grb2 is a key adaptor in the TGF-β2 > PI3-K signaling cascade.

The survival of *Theileria*-transformed cells depends on the constitutive activation of different transcription factors including AP-1^23^. In *Theileria*-infected macrophages there is a significant variation in PI3-K levels between virulent and attenuated cells, where PI3-K activity is augmented in aggressive, invasive virulent macrophages. However, PI3-K activity is decreased by ablation of Grb2 SH3 domain interactions by peptidimer-c treatment that also reduces AP-1 activity to levels equivalent to Wortmannin inhibition of PI3-K. In contrast, addition of TGF-β2 to attenuated macrophages restores PI3-K activity. All of the above is consistent with the notion that TGF-β2 stimulation of attenuated macrophages restores Grb2 expression, which results in recruitment of the p85 regulatory subunit of PI3-K proximal to TGF-RII and activation of PI3-K signaling and activation of AP-1 in *Theileria*-transformed macrophages.

JAM-L is implicated in adhesion of *Theileria*-infected macrophages^30^ and in another cellular system clustering of JAM-L activates PI3-K signaling^33^ suggesting a possible interaction occurs between JAM-L and PI3-K in *Theileria*-infected macrophages. Ablation of SH3 interactions of Grb2 by peptidimer-c, or inhibition of PI3-K signaling by Wortmannin both decreased mRNA levels of *JAM-L*. Furthermore, as *JAM-L* is an AP-1-target gene^30^ it suggests that PI3-K increases expression of *JAM-L* through AP-1 transactivation.

AP-1 complexes of altered mobilities can be observed in both *T. annulata* and *T. parva*-infected leukocytes, suggesting that parasite infection induces AP-1 heterodimers of different composition^41^. Previous examination of the same *Theileria*-transformed macrophages (Ode) as used here, revealed loss of binding to the *mmp9* promoter of a fast-migrating AP-1 family member and reduced MMP9 expression in attenuated Ode macrophages^38^. Here, using specific anti-Fos-B antibodies we identified the fast migrating AP-1 family member and treatment with peptidimer-c ablated Fos-B levels and AP-1 transactivation and consequently MMP9 expression in virulent *Theileria*-transformed macrophages. Virulent Ode macrophages treated with peptidimer-c resemble attenuated Ode macrophages generated by multiple *in vitro* passages.

**Figure 6:**
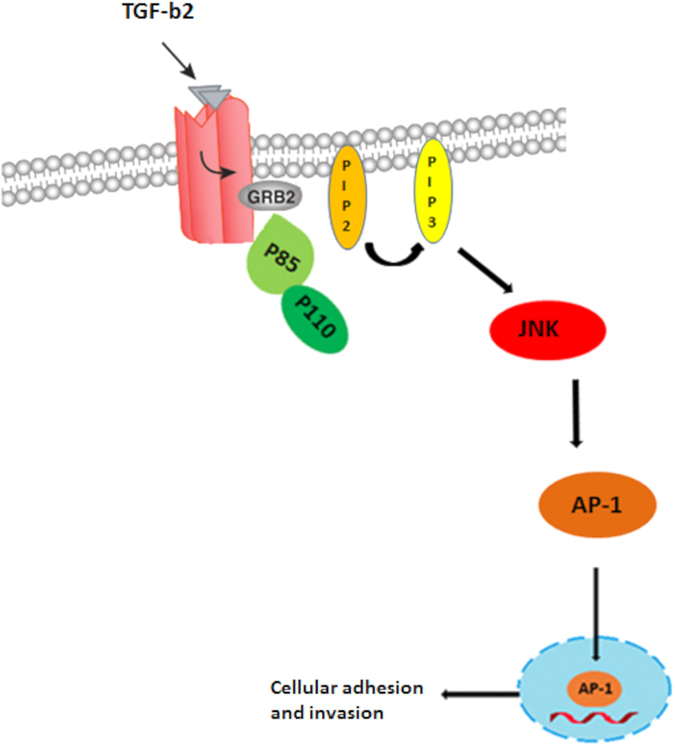
Pathway showing how TGF-β2 induction of Grb2 recruits PI3-K to TGF-RII to activate AP-1-driven gene transcription that underpins heightened adhesion and invasiveness of *Theileria*-transformed virulent macrophages.

In summary, we propose a scheme that explains how changes in expression of TGF-β2 orchestrate the switch from a virulent to an attenuated phenotype via Grb2 recruitment of PI3-K to TGF-RII that results in activation of AP-1 to regulate both adhesion and invasiveness of transformed macrophage (Fig. 6). The ensembles of our results indicate that peptidimer-c could eventually see use as a treatment for tropical theileriosis and perhaps even have applications in treatment of a wide range of cancers. Finally, as attenuated *Theileria*-infected macrophages display heightened oxidative stress that underpins their loss of adhesion/invasiveness^42^ it will be interesting to examine if loss of TGF-β2-signaling contributes to impaired regulation of oxidative stress in attenuated macrophages^42^. One possibility is that Grb2 binds catalase^43^ and its recruitment augments the conversion of H_2_O_2_ into H_2_O, so enabling *Theileria*-transformed leukocytes to better resists oxidative stress stemming for infection induced uncontrolled host cell proliferation^44^. Thus, future experiments will address the possible role of activation of the TGF-β2/Grb2/PI3-K/AP-1 pathway to regulation of oxidative stress in *Theileria*-transformed macrophages.

## Materials and Methods

### *Theileria annulata*-infected cell lines and cell culture

*T. annulata*-infected macrophages used in this study are the Ode and Jed4 virulent and attenuated vaccine lines^25^,^45^. Virulent (early passage) Ode corresponds to passage 62 and attenuated (late passage) Ode to passage 309, whereas virulent and attenuated Jed4 correspond to passages 18 and 327, respectively. All cells were incubated at 37 °C with 5% CO_2_ in Roswell Park Memorial Institute medium (RPMI) supplemented with 10% Fetal Bovine Serum (FBS), 2 mM L-Glutamine, 100 U penicillin, 0.1 mg/ml streptomycin, and 4-(2-hydroxyethyl)-1-piperazineethanesulfonic acid (HEPES).

### Ablation of the interaction of Grb2 and its partners

The SH3 interactions of Grb2 with its partners were ablated using a specific Grb2-SH3 competitive peptide (peptidimer-c)^29^,^31^. A concentration 1 μM was used and incubated for 1 h at 37 °C in 5% CO_2_.

### Activation of TGF-β-signaling pathway

The TGF-β signaling pathway was activated by using recombinant bovine TGF-β2 (rbo-TGF-β2) (NIBSC, Potters Bar. UK). A concentration of 2.5 ng/ml was added to the cell culture for 24 h.

### Total RNA extraction and reversed transcription

Total RNA of *Theileria*-infected macrophages was isolated using the RNeasy mini kit (Qiagen), according to the manufacturer’s instructions. The quality and quantity of RNA was measured by Nanodrop spectrophotometer. For the reverse transcription, 1 μg isolated RNA was diluted by water to a final volume of 12 μL, warmed at 65 °C for 10 min, then incubated on ice for 2 min. Afterwards, 8 μl of reaction solution (0.5 μL random hexamer, 4 μL 5× RT buffer, 1.5 μL 10 mM dNTP, 1 μL 200U/μLRT-MMLV (Promega) and 1 μL 40 U/μL RNase inhibitor (Promega) was added to get a final reaction volume of 20 μL and incubated at 37 °C for 2 h. The resultant RNA was stored at −20 °C.

### Quantitative polymerase chain reaction (qPCR)

mRNA expression levels were measured by qPCR on Light Cycler 480 (Roche) using SYBR Green detection (Thermo). *GAPDH* was used as internal control to normalize for mRNA levels. The detection of a single product was verified by dissociation curve analysis and relative quantities of mRNA calculated using the method described^46^. For list of primers used, see below (Table 1).

### Western blot analysis

Cells were washed with cold PBS and then lysed on ice for 30 min in lysis buffer (50 μL RIPA buffer 2X, 50 μL PBS). Cells were centrifuged at 13000 rpm for 15 min at 4 °C to eliminate cellular debris. Protein concentration was determined by using Bradford method (by reading optical density at 595 nm). Cell extract was mixed with 5X Laemmli and denatured at 95 °C for 5 min. 10 μg of protein were separated by migration through a denaturing 10% SDS-PAGE gel and electro-transferred onto a nitrocellulose membrane (Protan). The membrane was blocked by 5% non-fat milk-TBST (for antibody of interest), 3% non-fat milk-PBST (for anti-Actin antibody) for 1 h at RT. Antibodies used were as follows: mouse monoclonal antibody anti-Grb-2 (Santa Cruz Biotechnology #8034) diluted 1/1000 in 5% BSA-TBST, rabbit polyclonal antibody anti-JNK (Santa Cruz Biotechnology #571) diluted 1/1000 in 5% BSA-TBST, rabbit polyclonal antibody anti-p85 (Santa Cruz Biotechnology) diluted 1/1000 in 5% BSA-TBST and mouse monoclonal antibody anti- Phospho-SAPK/JNK (Thr183/Tyr185) (Cell signaling #9255) diluted 1/1000 in 5% BSA-TBST all incubated overnight at 4 °C. Goat polyclonal antibody anti-Actin (Santa Cruz biotechnology, I-19) diluted by 1/2000 in 3% BSA-PBST was incubated for 1 h at RT. After washing, membranes were incubated with peroxidase-conjugated secondary antibody (mouse anti-IgG, rabbit anti-IgG and goat anti-IgG (Santa Cruz biotechnology) diluted by 1/5000 for 1 h at RT. After washing, membranes were developed using Super Signal detection kit (Thermo).

### GST-Grb2 pull downs

Cells were washed with cold PBS and then lysed on ice for 30 min in lysis buffer (50 μL RIPA buffer 2X, 50 μL PBS). The cells were centrifuged at 13000 rpm for 15 min at 4 °C to eliminate cellular debris. 50 μl of cell lysate was collected and 10 μl of GST-Grb2 beads and incubated over night with gentle rocking at 4 °C. Cell extract was mixed with 2X Laemmli and denatured at 95 °C for 5 min. Proteins were separated by migration through a denaturing 8% SDS-PAGE gel and electro-transferred onto a nitrocellulose membrane (Protan). The membrane was blocked by 5% non-fat milk-TBST for 1 h at RT. The proteins were analyzed using a rabbit polyclonal primary antibody anti-TGFβ RII (Santa Cruz biotechnology #sc-220-R) diluted by 1/200 5% BSA-TBST and a rabbit polyclonal primary antibody anti-p85 both incubated overnight at 4 °C. After washing, membranes were incubated with peroxidase-conjugated secondary antibody rabbit anti-IgG (Santa Cruz biotechnology #2005) diluted by 1/5000 in 5% non-fat milk-TBST for 1 h at RT. After washing, membranes were developed using Super Signal detection kit (Thermo).

### AP-1-luciferase assay

Ode macrophages were transfected by electroporation using the Amaxa Nucleofector, kit V and program T -017 (Amaxa Lonza protocol). 5 × 10^5^ cells/ml were washed once with PBS at room temperature, and then cells were suspended in 100 μl of Nucleofector solution V (Amaxa). Cells were co-transfected with 2 μg of luciferase reporter plasmid (3X-TRE) and/or reporter plasmid (β-galactosidase: β-gal). After transfection, cells were suspended in fresh complete medium and incubated at 37 °C, 5% CO_2_ for 24 h. Measurements of luciferase and β-galactosidase activities were performed using the Dual Light Assay system (Life Technologies) and luminometer Centro LB 960 (Berthold) according to the manufacturer’s instructions.

### PI3-Kinase activity

Cells were grown to 80–90% confluence in a 6-well plate, pre-treated with peptidimer-c (1 μM for 1 h) and TGF-β2 (5 ng/ml for 30 min) and incubated at 37 °C. Extraction of PIP_3_ from cells followed the protocol in the PIP_3_ Mass ELISA kit (Echelon #k-2500 s). PIP_3_ levels were estimated using the PI3-K activity/inhibitor assay kit (Millipore #17–493) according to the manufacturer’s instructions. Briefly, the kit contains recombinant protein GRP-1, which binds to the glutathione plate and captures either PIP_3_ generated by the PI3-kinase reaction, or the biotinylated-PIP_3_ tracer included in the kit. The captured biotinylated-PIP3 is detected using streptavidin-HRP conjugate and following colorimetric development the optical density was measured at 405 nm.

### Dynamic monitoring of cell adhesion: xCELLigence (Roche)

Fibronectin (0.7 μg/ml) was added to wells on 96X E-plate then the plate incubated for 30 min at 37 °C. The protein-coated plates were washed with PBS and incubated with 0.5% BSA solution in PBS for 1 h at 4 °C. The wells of the treated plates were washed with PBS. 50 μL of the cell suspension was transferred to wells on E-plates. The extent of cell adhesion and spreading, was monitored every 5 min for a period of 2 h. The xCELLigence System monitors cellular events in real time without the incorporation of labels. The System measures electrical impedance across interdigitated micro-electrodes integrated on the bottom of tissue culture E-Plates.

### Matrigel chambers assay

The invasive capacity of Ode macrophages was assessed *in vitro* using Matrigel migration chambers, as described^47^. Culture coat 96-well medium BME cell invasion assay was obtained from Culturex instructions (3482-096-K). After 24 h of incubation at 37 °C, each well of the top chamber was washed once in buffer. The top chamber was placed back on the receiver plate. 100 μL of cell dissociation solution/Calcein AM were added to the bottom chamber of each well, incubated at 37 °C for 1 h to fluorescently label cells and dissociate them from the membrane before reading at 485 nm excitation, 520 nm emission using the same parameters as the standard curve.

### Statistical analysis

All experiments were repeated at least three times and the results were expressed as mean ± standard error (SE). The statistical analysis were calculated using Student’s t-test. Differences were considered significant when P < 0.05.

## Additional Information

**How to cite this article**: Haidar, M. *et al*. TGF-β2 induces Grb2 to recruit PI3-K to TGF-RII that activates JNK/AP-1-signaling and augments invasiveness of *Theileria*-transformed macrophages. *Sci. Rep*. **5**, 15688; doi: 10.1038/srep15688 (2015).

## Figures and Tables

**Table 1:** List of primers

	**Forward**	**Reverse**
*GAPDH*	AGGACAAAGCTCAGGGACAC	CCCCAGGTCTACATGTTCCA
*Grb2*	CCAGGTTCAGCACTTCAAGG	CTGGGCCGAGAAGTCAAAC
*JAM-L*	GATTGTCTGCACCACCAT	AGGTGGCCTCTGATTTTCC
